# Identifying robust communities and multi-community nodes by combining top-down and bottom-up approaches to clustering

**DOI:** 10.1038/srep16361

**Published:** 2015-11-09

**Authors:** Chris Gaiteri, Mingming Chen, Boleslaw Szymanski, Konstantin Kuzmin, Jierui Xie, Changkyu Lee, Timothy Blanche, Elias Chaibub Neto, Su-Chun Huang, Thomas Grabowski, Tara Madhyastha, Vitalina Komashko

**Affiliations:** 1 Rush University Medical Center, Alzheimer’s Disease Center, Chicago, IL; 2 Allen Institute for Brain Science, Modeling, Analysis and Theory Group, Seattle, WA; 3 Rennselaer Polytechnic Institute, Department of Computer Science, Troy, NY; 4 Społeczna Akademia Nauk, Łódź, Poland; 5 Samsung Research America, San Jose, CA; 6 Sage Bionetworks, Seattle, WA; 7 University of Washington, Department of Neurology, Seattle, WA; 8 University of Washington, Department of Radiology, Seattle, WA; 9 Trialomics, Seattle WA

## Abstract

Biological functions are carried out by groups of interacting molecules, cells or tissues, known as communities. Membership in these communities may overlap when biological components are involved in multiple functions. However, traditional clustering methods detect non-overlapping communities. These detected communities may also be unstable and difficult to replicate, because traditional methods are sensitive to noise and parameter settings. These aspects of traditional clustering methods limit our ability to detect biological communities, and therefore our ability to understand biological functions. To address these limitations and detect robust overlapping biological communities, we propose an unorthodox clustering method called SpeakEasy which identifies communities using top-down and bottom-up approaches simultaneously. Specifically, nodes join communities based on their local connections, as well as global information about the network structure. This method can quantify the stability of each community, automatically identify the number of communities, and quickly cluster networks with hundreds of thousands of nodes. SpeakEasy shows top performance on synthetic clustering benchmarks and accurately identifies meaningful biological communities in a range of datasets, including: gene microarrays, protein interactions, sorted cell populations, electrophysiology and fMRI brain imaging.

Molecules, cells and tissues carry out biological processes through physical interaction networks^1^,^2^,^3^ and can enter disease states when those networks are disrupted^4^,^5^,^6^,^7^. Because the structure of networks is related to the functions they carry out^8^,^9^, it is possible to investigate biological functions by examining network structure^3^,^10^,^11^,^12^,^13^,^14^. Densely connected groups known as communities are prevalent in biological networks and may be related to specific molecular, cellular or tissue functions^10^,^15^,^16^,^17^. Therefore, biological community detection is a key first step in many network-based biological investigations. However, accurately identifying biological communities is challenging, because network structures often have incorrect or missing links, because traditional methods can produce unstable results^18^,^19^, and because biological communities tend to be highly overlapping^20^,^21^,^22^.

## SpeakEasy: A new label propagation algorithm to detect overlapping clusters

We propose a label propagation clustering algorithm, “SpeakEasy”, to robustly detect both overlapping and non-overlapping (disjoint) clusters in biological networks. SpeakEasy is related to earlier label propagation algorithms^23^,^24^,^25^ in the sense that nodes join communities based on exchange of “labels” between connected nodes. These “labels” do not refer to *a priori* community titles. In this context, labels are unique bits of information that are assigned randomly and used to track cluster membership. SpeakEasy differs from previous label propagation algorithms, because nodes update their labels on the basis of their neighbors’ labels, while subtracting the expected frequency of these labels, based on their popularity in the complete network. This process combines a bottom-up approach to clustering (using neighboring information) with a top-down approach (using information from the whole network). This dual approach facilitates accurate community detection in many types of biological networks (Table 1) because top-down information is used to ensure the bottom-up label propagation process identifies communities that accurately represent the global network structure^19^,^26^,^27^,^28^.

In addition to accurate cluster detection (see Results section), community detection via SpeakEasy has several practical advantages for biological applications. For instance, since the number of communities in a dataset is rarely known in advance, SpeakEasy automatically predicts the number of communities and does *not* require manual tuning of clustering parameters for good results. Second, it can cluster networks with any type of links (weighted/unweighted, directed/undirected, positive/negative-valued edges) or any type of network structure (networks with several different degree distributions). SpeakEasy is highly scalable and can quickly cluster networks with hundreds of thousands of nodes. Third, because it is very efficient, the stochastic clustering process can be repeated many times to detect robust clusters that are not generated by data artifacts or noise. The repeated clustering process also allows SpeakEasy to identify multi-community nodes, whose membership tends to oscillate between different clusters. Finally, users can select overlapping or non-overlapping output, as is appropriate for their applications.

## Visual example of SpeakEasy clustering

**Figure 1:**
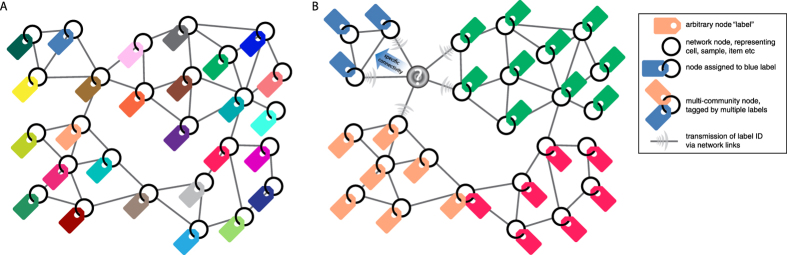
Intuitive schematic of the core SpeakEasy clustering mechanism. (**A**) Clusters are determined by competition between nodes through “labels” (symbolized here by colored tags) that grow and spread through a network. (**B**) SpeakEasy groups nodes according to the communities to which they are most specifically connected. Thus, when nodes connected to the gray node broadcast their identities, it will join the “blue” community on the upper left, because its connectivity to more popular labels is expected at random. Nodes are classified as multi-community nodes if they fit equally well with multiple communities (for example, the node tagged with both orange and red labels, see methods for details). Technical details of the algorithm are provided in the methods section and pseudocode for the complete algorithm is provided in the Supplementary text.

For an intuitive example of how SpeakEasy identifies communities, we illustrate the clustering process on a demonstration network (Fig. 1A). This network can represent any type of biological component, such as genes, proteins or tissues; network links could be derived from primary data or scientific literature. Initially, labels (represented by colored tags) are applied randomly to all nodes (Fig. 1A), with the total number of labels equal to the total number of nodes. Then, each node updates its label, based on the labels of neighboring nodes. Specifically, a node will adopt the label found most commonly on its neighbors taking into account the global frequency of all labels (i.e., it will adopt the label that is most specific to its neighbors). For instance, the node shown in gray (Fig. 1B) is connected to orange-, blue- or green-labeled communities, so it must adopt one of these three labels. The gray node will update its label to the blue tag, because it has the strongest specific connection to the blue community, even though it has an equal number of links to the green community. Through this updating process, densely connected groups of nodes will acquire the same label. Multi-community nodes tend to oscillate their membership between multiple communities, such as the node located between the red and orange communities (Fig. 1B). The complete algorithm is described in the methods and in the supplement via pseudocode.

## Results

### Summary

We use three approaches to determine the accuracy of SpeakEasy community detection. First, we test its performance on a large set of synthetic networks with carefully controlled characteristics, wherein the true clusters are known. Then we apply it to real-world networks, wherein the true clusters are unknown (Table 2). In this second context we can quantify community detection accuracy by using the statistical separation between clusters. Finally, we apply SpeakEasy to several types of common biological networks (Table 1). This collection of applications was selected because they have multiple of the following characteristics: 1) analysis of these datasets often utilizes clustering; 2) they have high levels of noise; 3) they are generated via different technologies measuring biological properties at several physical scales; 4) they can benefit from overlapping community detection, and 5) their true community structure is unknown or debated. In all cases, we make comparisons to alternate methods that have been applied to the same or similar datasets.

### Synthetic clustering benchmarks

**Figure 2:**
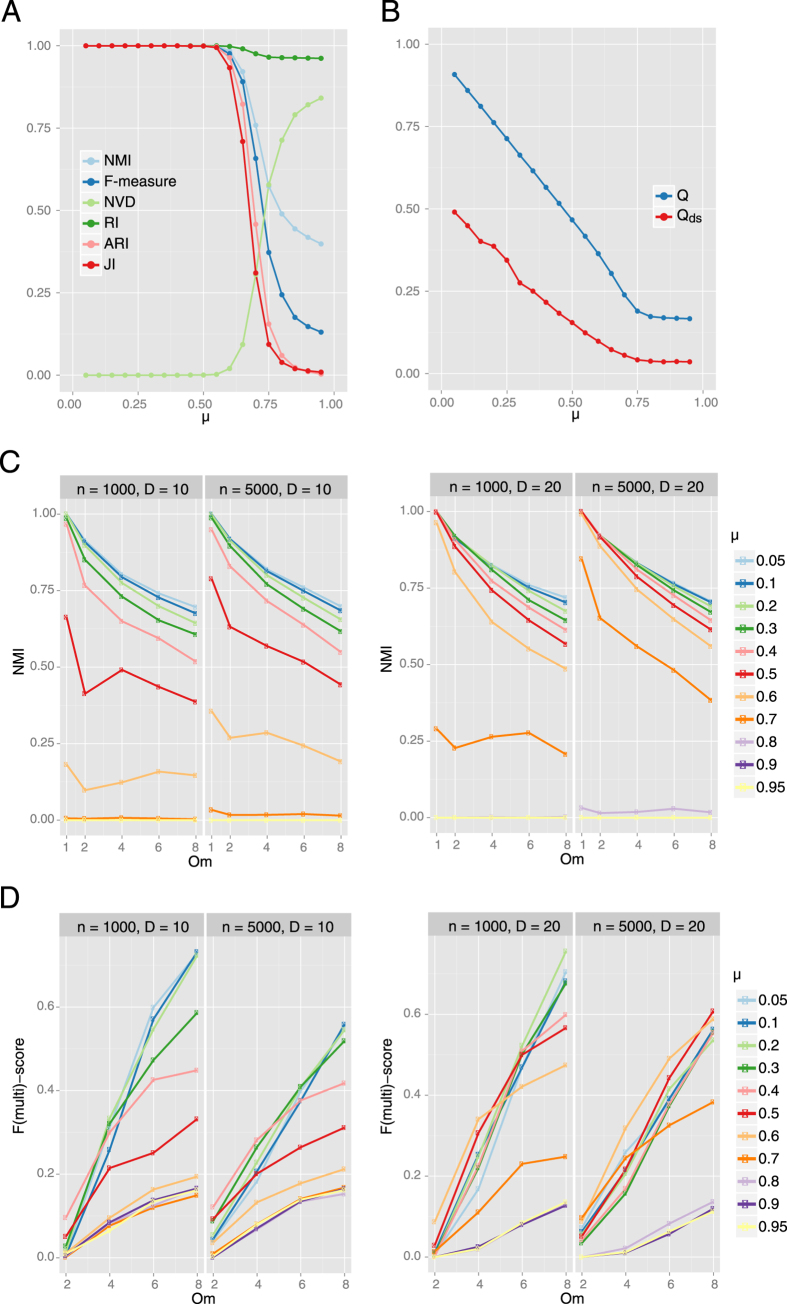
Disjoint cluster detection performance. (**A**) The LFR benchmarks track cluster recovery as networks become increasingly cross-linked (as μ increases) for γ (cluster size distribution parameter) equal to 2 and β (within-cluster degree distribution parameter) equal to 1. Several metrics characterize cluster recovery with varying levels of sensitivity. For the following measures (min = 0), lower values indicate better alignment between the true partition and partition generated by SpeakEasy: NVD - Normalized Van Dongen metric. For the following measures, larger values (max = 1) indicate better alignment between the true and SpeakEasy partitions: NMI - Normalized Mutual Information; F-measure; RI- Rand Index; ARI - Adjusted Rand Index; JI - Jaccard Index. See Chen *et al.*^34^ for additional details on these statistical measures. (**B**) These modularity values provide a statistical estimate of the separation between clusters. For both Q (modularity) and Q_ds_ (modularity density), larger values (max = 1) indicate better community separation. (**C**) Recovery of true clusters quantified by NMI as a function of μ (cross-linking between clusters) and O_m_ (number of communities associated with each multi-community node). (**D**) F(multi)-score is the standard F-score, but specifically applied for detection of correct community associations of multi-community nodes, calculated at various values of O_m_ and different average connectivity levels (D = 10,20). NMI metric used for overlapping communities (panels **C,D**) does not reduce to disjoint NMI, so NMI scores for O_m_ = 1, cannot be directly compared to panel A.

To generate networks with known community structure, we use the Lancichinetti-Fortunato-Radicchi (LFR) benchmarks, which are widely used to test overlapping and non-overlapping clustering methods^29^. These benchmarks contain a range of networks, some with well-separated clusters and other networks with clusters that are highly cross-linked and almost indistinguishable. We track the accuracy of communities detected by SpeakEasy under increasing levels of cross-linking (μ) (Fig. 2A), using average results from 10 replicate runs at each parameter setting. The effect of cross-linking (increasing μ) is reflected by decreasing modularity (Q) and modularity density (Q_ds_) (Fig. 2B). SpeakEasy shows the highest-yet accuracy in community detection, based on normalized mutual information (NMI)^25^,^30^,^31^,^32^,^33^, especially for highly cross-linked clusters (μ = 0.95) (Fig. 2A). Additional cluster recovery statistics such as the adjusted Rand index have varying inputs and sensitivity^34^, but also support this strong ability to detect true communities. While NMI is the most common way to report comparisons to known clusters, some of these additional metric may be relevant, as specific biological experiments may place different weight on false positive or false negative results. These results are not affected by various distributions of cluster size or intra-cluster degree distributions (Figure S1). Thus, SpeakEasy can accurately identify disjoint clusters in the most popular clustering benchmarks, even when these clusters are heavily obscured by cross-linking/noise.

We also test community detection on LFR networks with overlapping communities. In this setting, SpeakEasy also shows excellent community detection performance and the ability to identify multi-community nodes (Fig. 2C,D)^35^. As seen previously for disjoint networks (Fig. 2A), increasing the level of cluster cross-linking (μ) makes community detection more challenging, resulting in lower NMI with the true set of clusters. Better community detection accuracy was achieved for networks with higher average connectivity (D). This can be explained by the greater cluster density of these networks (Fig. 2). Community detection is also affected by the number of communities that are tied to multi-community nodes (O_m_). When multi-community nodes are tied to many communities (high O_m_ values), community detection becomes more difficult (Fig. 2C,D). This response to highly overlapping communities is universal across overlapping clustering algorithms^35^. Community detection scores for most methods also tend to decrease on large networks^35^. This decrease in performance could be more severe for SpeakEasy, because it employs a diffusion process. However, SpeakEasy performs slightly better on networks of 5000 nodes versus networks with 1000 nodes. This may be explained by the incorporation of global network information (label popularity) into the local clustering process^26^,^27^,^28^.

### Abstract clustering performance on diverse real-world networks

The LFR benchmarks accurately represent certain aspects of social and biological networks, but are limited in other aspects. For example, networks in the LFR benchmarks have low transitivity and null assortativity (propensity for hubs to connect to hubs)^36^. Therefore we apply SpeakEasy to fifteen real networks that are often used to test clustering methods. Unlike the LFR benchmarks, the true community memberships in these networks are unknown. However, the quality of clusters detected by various methods can be compared by using modularity (Q)^37^ and modularity density scores (Q_ds_)^38^, which quantify how well a given network is segmented into dense clusters.

We compare modularity values from SpeakEasy to those from another label propagation algorithm, GANXiS, because that method showed the best overlapping clustering performance in a recent comparison of clustering methods^35^. In this comparison, SpeakEasy shows improved performance on 6 out of 15 networks using the modularity (Q) metric, with a mean percent difference in performance of 2% over GANXiS (Table 2). Using density based Q_ds_ metric that was shown to be more consistent with other metrics than original Q metric^38^,^39^, SpeakEasy performs better than GANXiS on 14 out of 15 networks with a mean percent difference of 28% over GANXiS (see Supplementary Materials). The consistently high Q_ds_ values from SpeakEasy (compared to Q-values) indicate that it tends to detect more small and highly dense clusters than GANXiS^38^. SpeakEasy shows both higher Q and Q_ds_ scores for the two biological networks in this test set (‘dolphins’ and ‘c.elegans’). These modularity values are approach those of methods that directly attempt to maximize modularity^34^. Consistently high modularity on networks of diverse origin indicates that a simultaneous top-down and bottom-up approach to clustering functions will succeed on a wide range of topologies. However, high modularity is still not a proof of real utility in clustering biological networks. Therefore, we apply SpeakEasy to several types of biological networks, and compare the output clusters to gold-standards or to literature-based ontologies.

### Application to protein-protein interaction datasets

**Figure 3:**
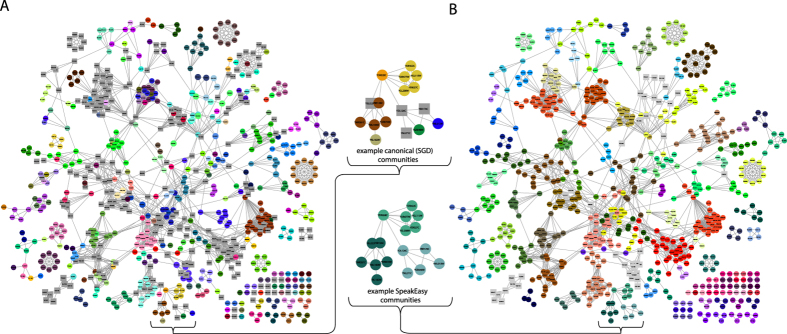
Contrasting protein complex membership, estimated by small-scale experiments and high-throughput clustering. (**A**) The high throughput interaction dataset from Gavin *et al.*^40^ has nodes colored according to complexes found in the Saccharomyces Genome Database (SGD) database. Nodes found in multiple protein complexes are shown as gray squares. (**B**) The clusters identified by SpeakEasy are color-coded. Nodes found in multiple communities are depicted as gray squares. Inset: network fragments show example positions of actual versus inferred multi-community nodes in a portion of the network, showing how some canonical multi-community nodes have very little support for that classification, based on the network structure.

Because a single protein may be part of more than one protein complex (set of bound proteins that work as a unit), Discovery of protein complexes directly benefits from development of methods which detect overlapping communities. We test SpeakEasy community detection of overlapping protein complexes, using two well-studied high-throughput protein interaction networks (Gavin *et al.*^40^ and Collins *et al.*^41^) derived from affinity purification and mass spectrometry (AP-MS) techniques. We then compare the predicted clusters against three gold-standards for protein complexes^42^,^43^,^44^ (Fig. 3). NMI scores between the predicted and the true protein complexes indicate that SpeakEasy produces the most accurate recovery of protein complexes to date^32^,^33^,^45^ (Table 3). We also examine precision and recall statistics specifically for the detection of multi-community nodes. SpeakEasy identifies a smaller number of multi-community nodes than are listed in various gold-standards, although the multi-community nodes it does detect are often in agreement with the gold-standards (Table 3). However, there may be upper limits on using the Collins and Gavin datasets to measure multi-community node detection, because there is frequently no evidence (links) in these networks in support of canonical multi-community nodes (Fig. 3 inset).

### Application to cell-type clustering

**Figure 4:**
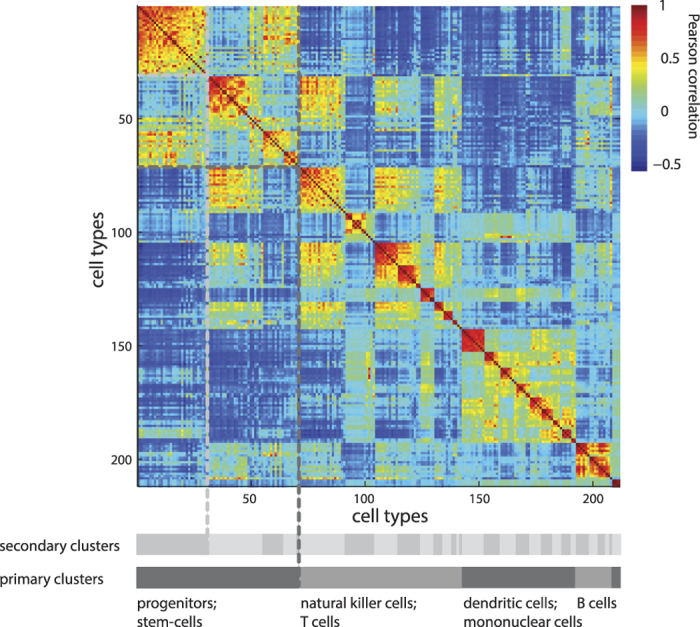
Primary and secondary biological classifications of immune cell types are reflected in primary and secondary clusters. The clustered correlation matrix of similarity of cell expression vectors is ordered according to primary clusters, which correspond to large-scale cell families such as B-cells, and secondary clusters, which correspond more closely to a more detailed classification of the intersection of cell-type and tissue of origin (see also Table 4).

Identifying robust cell populations that constitute a true cell type is a challenging problem, due to ever-increasing levels of detail on cellular diversity. To explore how traditional clustering methods and SpeakEasy can be used to identify robust cell-types, we use a collection of sorted cell populations from the Immunologic Genome Project (Immgen)^46^,^47^. The immune system contains many populations of cells that can be distinguished by specific combinations of cell surface markers as well as broader functional families, such as dendritic cells, macrophages and natural killer cells. We apply SpeakEasy to a matrix of expression similarity from cells from 212 cell types, as defined in Immgen. We then compare our results with the primary classification of the sorted cells. There is a strong correspondence between the identified clusters and the tissue origin of these cells. (Fig. 4, Table 4).

We find that applying SpeakEasy once again, to each of these broad categories of cell types, identifies sub-communities with higher correspondence to the tissue of origin and cell type, considered together (Table 4). Thus, successive applications of SpeakEasy clustering results may reflect successive tiers of biological organization. In comparison to standard hierarchical clustering methods, even when those methods are supplied with the true number of clusters, SpeakEasy still shows the highest correspondences with canonical cell types (see Supplementary Materials). These results indicate SpeakEasy will be useful in future applications, where the number of communities (in this case, cell types) is unknown.

### Application to finding coexpressed gene sets

Several cellular or molecular processes can generate correlated gene expression (called coexpression), including cell-type variation, transcription factors, epigenetic or chromosome configuration^48^. Identifying genes which are coexpressed in microarray or RNAseq datasets is useful because these gene sets may carry out some collective functions related to disease or other phenotypes. This task is challenging because coexpressed genes may be context-specific and therefore lack gold-standards, gene expression data tends to be noisy, and these gene sets are generated by overlapping mechanisms^21^,^49^.

Therefore, we use SpeakEasy to detect overlapping and non-overlapping coexpressed gene sets in two datasets that are commonly used to address many biological questions: The Human Brain Atlas (HBA)^50^, comprised of 3584 microarrays measured in 232 brain regions and the Cancer Cell Line Encyclopedia (CCLE)^51^, comprised of 1037 microarrays from tumors found in all major organs. We find 40 non-overlapping clusters in HBA containing more than 30 genes (a practical threshold to assess functional enrichment), with a median membership of 384 (see Supplementary Materials). In CCLE we find 43 clusters with more than 30 gene members, with a median community size of 265. Coexpressed gene sets tend to be involved in certain biological functions; therefore, these gene sets tend to have high functional enrichment scores based on ontology databases such as Gene Ontology (GO) and Biocarta [50]. Of these 40 large clusters we detect in HBA, 27 have an average Bonferroni-adjusted p-value of <0.01 for one or more biological processes. Of the 43 large clusters we detect in CCLE, 35 have a Bonferronni-adjusted p-value of <0.01.

We also generate overlapping clusters from both the HBA and CCLE datasets. Overlapping coexpressed gene sets may be useful in biological studies because gene coexpression is driven by overlapping mechanisms^21^. Furthermore, assigning truly multi-community nodes to only a single community will produce inherently inaccurate communities. When multi-community SpeakEasy output is enabled, we still detect 40 clusters in HBA data, but the median size increases from 384 to 544, with 4510 genes holding overlapping community membership. Overlapping results from CCLE show an increase in median module size from 265 (non-overlapping) to 702, with ~10,000 genes found in more than one community. Functional enrichment scores for overlapping HBA gene sets are equivalent to non-overlapping results, while enrichment scores for gene sets from CCLE were several orders of magnitude more significant. We conduct a comparison of these results to the WGCNA method commonly used to identify coexpressed genes (see Supplementary Material), which shows practical benefits of SpeakEasy, including higher functional enrichment and avoiding of arbitrary filters and complex parameter settings.

### Application to neuronal spike sorting

Extracellular neuronal recording with single electrodes, tetrodes, or high density multichannel electrode arrays can detect the activity of multiple nearby neurons. However, these combined responses must be separated into responses of specific neurons. This blind source separation process is known as “spike sorting”, because each spike is assigned to a particular theorized neuron. Single neurons often generate relatively unique signatures (i.e. spike waveform shapes and amplitude distributions on multiple adjacent electrodes), and emerge as clusters in the matrix of waveform correlations.

To realistically test spike sorting, it is important to match noise levels in real brain recordings. Therefore, we use real depth-electrode recordings generate a simulated time-series of spikes in which the true spike times and unique neuronal sources are known (see Supplementary Materials). Comparison of the inferred clusters (represent the activity of a single neuron) to the true associations between spikes and neurons indicates that SpeakEasy can reliably sort spikes from multielectrode recordings (Table S1). The waveforms associated with each cluster can then be used in template-matching to detect additional spikes from the same neuronal origin.

### Application to resting-state fMRI data

Functional magnetic resonance imaging (fMRI), obtained while a subject is at rest (rs-fMRI), is a valuable tool in understanding of systems-level changes in a variety of domains, including neurodegenerative disease^52^. Correlations between the rs-fMRI signals in different regions of interest (ROIs) may indicate which regions are functionally related. Brain networks composed of functionally-related ROI’s tend to be noisy and overlapping because ROIs perform functions for multiple networks or because the low temporal resolution of the blood oxygen level-dependent signal causes temporal smearing of brain networks. The ability to robustly identify functional networks (communities), and changes to this structure that occur with disease, is critical to understanding the physiological changes that may be early indicators of disrupted cognitive function.

**Figure 5:**
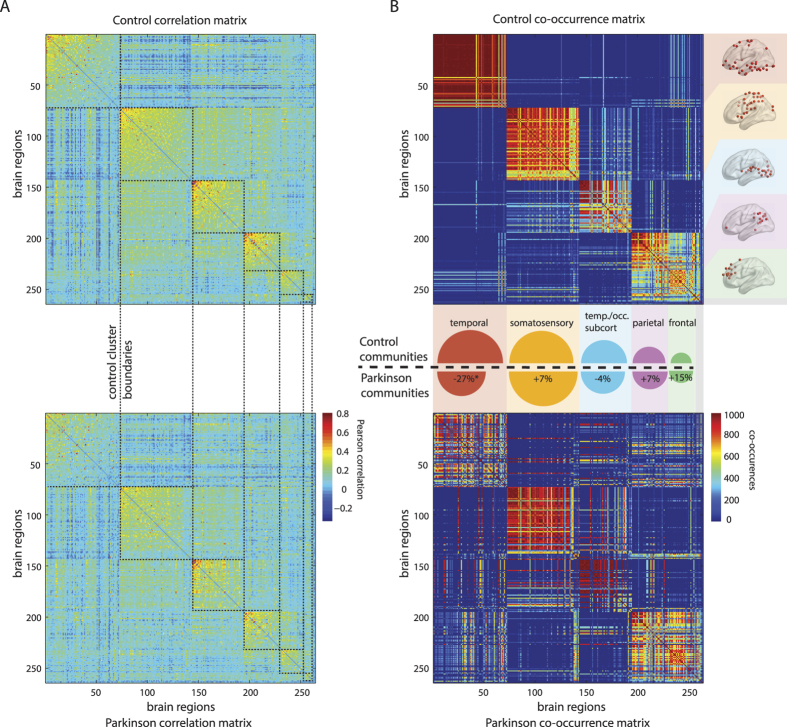
Shifts within and between resting-state brain communities in Parkinson disease. (**A**) Raw correlation matrices between resting state brain activity from control and Parkinson disease cohorts. Dashed lines indicate clusters identified by SpeakEasy from control-state data. Order of brain regions is identical in all matrices (reflects control-state clusters). (**B**) Co-occurrence matrices for controls and Parkinson disease cohorts. Entries in co-occurrence matrices count the number of times nodes (i,j) are found together in 100 replicated clustering results. (Inset) Semi-circles are scaled by volume to cluster size in control data. The *difference* in size of the corresponding lower semi-circles illustrates the change in average co-occurrence for each control-state cluster. Thus smaller semi-circles in disease (lower half) denote loss of coherence among members of a particular cluster. Text in semi-circles summarizes the most common regional characteristic of each cluster.

Figure 5A shows the relatively small inter-regional correlations characteristic of rs-fMRI functional connectivity graphs in control subjects (n = 21) and subjects with Parkinson disease (PD, n = 27)^53^ (Table S2). Due to high levels of noise and weak community structure (Fig. 5A), apparent communities of brain regions may easily be driven by clustering parameters or data artifacts. Therefore, we apply SpeakEasy to the average control and PD rs-fMRI connectivity matrices 1000 times, to quantify the stability of each cluster through co-occurrence matrices (Fig. 5B). For instance, in control subjects, the community of temporal areas is very stable (has high average co-occurrence) while the cluster of parietal areas is less stable. We then use a permutation test to identify communities of brain regions that change their membership between control and PD groups (see Supplementary Materials).

Communities identified in control and PD groups contain biologically similar sets of brain regions (quantified in Table S3), but specific communities alter their membership significantly in PD. Using clusters from control subjects as a frame of reference, we observe both significant changes in community size and inter-community connectivity (see Supplementary Materials). A cluster comprised of (predominantly) temporal cortex ROIs showed the largest drop (−27%) in average co-occurrence among its members in PD (p<0.001). Specifically, the temporal cluster disintegrated in PD, with its area-members joining different communities (Fig. 5B). In PD subjects, the putamen and thalamus regions form an independent cluster in PD that is not observed in the control subjects, wherein those regions are part of the third largest cluster that is composed of temporal and occipital locations regions. Comparing these results to the alternative clustering method, Infomap^54^, which has been used previously with fMRI data^55^, show that method is sensitive to arbitrary link thresholds that it requires (see Supplementary Materials and Table S3). This sensitivity to parameter settings observed for InfoMap, is especially deleterious for noisy networks, such as those extracted from fMRI data. This situation, which likely leads to unstable or irreproducible clusters, can be avoided by using SpeakEasy to both generate robust results and to quantify the stability of each cluster, as we have demonstrated (Fig. 5).

## Discussion

Biological communities are a common feature of biological networks^9^,^10^ and are associated with execution of various cellular and molecular functions^12^,^14^,^15^,^56^. Therefore, identifying these communities with clustering methods is often the first step in understanding biological datasets. An ideal clustering algorithm should identify correct clusters in a synthetic setting and have excellent modularity results when true communities are unknown. Moreover, it should run in a reasonable time on large networks using standard hardware and without the need to manually “tune” method parameters for good results. When applied to biological networks, it should function well regardless of the type of data or particular network properties of the dataset. Finally these results should be robust and not driven by noise or method parameters. The performance of SpeakEasy on comprehensive biological tests indicates is fulfills these criteria.

Using a wide range of networks (Table 2) SpeakEasy produces higher modularity density scores than the best performing overlapping clustering method to date^25^,^35^. It has excellent absolute and relative performance on the LFR benchmarks (Fig. 2), scales well and can quickly cluster networks with hundreds of thousands of nodes on a typical laptop (Table 1 and Supplementary Materials). When applied to biological networks generated by distinct experimental methods, SpeakEasy detects robust, plausible, well-validated clusters (Figs 3, 4, 5, Tables 3, 4, Supplementary Materials). Collectively these results point to future potential for robust disjoint and overlapping clustering in related applications.

The extent to which the performance of SpeakEasy has a practical effect on biological results can be observed by comparisons to popular methods, in situations where the true biological communities are known or when they can be estimated. For instance, in the application of identifying groups of similar cell types, standard hierarchical methods generally have lower concordance with the true groups than does SpeakEasy (Table 4). This is the case even when hierarchical methods are provided with the correct number of clusters – which is rarely known in advance. InfoMap has previously been used to identify clusters of brain regions in fMRI data, but application to a similar dataset here indicates such results can be sensitive to clustering parameter settings (Table S3). Sometimes the stability of a specific cluster, rather than the overall clustering, is a practical concern in designing biological experiments. It is possible to estimate the stability of each cluster provided by SpeakEasy (Fig. 5B). Orthogonal data sources can also be used to quantify the goodness of specific clusters. For instance, we apply gene ontology enrichment tests to each cluster detected by SpeakEasy and an alternative algorithm (WGCNA), when applied to several coexpression datasets. The overall extent and significance of the enriched clusters is greater than or equal to those for the alternative WGCNA. Across all comparisons to popular methods applied to real biological datasets, SpeakEasy shows practically relevant advantages in cluster detection, due to a stable consensus approach.

The SpeakEasy algorithm could potentially be improved by changing how node labels are updated. Currently, nodes are updated to reflect the single most unexpected label among their neighbors. However, each node could be simultaneously characterized by multiple unexpected labels. This might aid in the identification of multi-community nodes or completely nested networks. In addition, binomial or multinomial tests may provide more accurate metrics for the unexpectedness of a given label. However, this altered label selection would not extend easily to weighted networks or networks with negative link weights. Selecting an updated label from a randomly chosen subset of inputs could improve results, as analogous improvements have been observed in Bayesian network inference when nodes have greater freedom to reconfigure their local network^57^. With these potential modifications, care must be taken to ensure that the network still converges to a clustered solution and does not become chaotic. SpeakEasy could also be improved by altering the consensus clustering routine used to identify the final partition and multi-community nodes. This consensus clustering step is completely separable from the label propagation process. Therefore, improvements to consensus clustering method could improve the overall results of SpeakEasy. An ideal consensus clustering method would quickly refine the structure of all of the clusters, using all partitions and output disjoint or overlapping clusters. However, few available techniques meet these criteria and consensus cluster methods are often slower than primary clustering methods^18^,^58^,^59^.

While SpeakEasy shows top performance among other available methods on multiple benchmarks and biological datasets, some alternative algorithms produce more accurate results for high O_m_ values on the LFR benchmarks^35^. However, the exact structure of a network is typically unknown in advance of clustering. Therefore, the generally excellent performance of SpeakEasy across many simulated and real networks indicates it will likely produce useful results on many datasets in the future.

## Methods

### Synthetic network benchmarks

To robustly measure the ability of SpeakEasy to recover true clusters from a range of network structures in the LFR benchmarks, we vary network characteristics (Fig. 2, Figure S1) including number of nodes, density of connections, distribution of cluster sizes, cluster separation and number of overlapping communities (see Supplementary Materials).

### Algorithm overview

An implementation of the SpeakEasy algorithm is provided free for non-commercial use here: http://www.cs.rpi.edu/~szymansk/SpeakEasy and it is also presented in pseudo-code (see Supplementary Materials). In summary, initially each node is assigned a random unique label. Then for some small number of iterations (usually less than 30), each node updates its status to the label found among nodes connected to it which has the greatest specificity, i.e. the label with the greatest difference between the actual and the expected frequency (Fig. 1 and Supplementary Materials). Positively or negatively-weighted links between nodes (often produced when clustering correlation-based networks) are easily incorporated into SpeakEasy, as they provide relative increases or decreases in the popularity of a particular label. The label updating step is performed simultaneously for all nodes. Although there is the potential for oscillating states to emerge with a simultaneous update step, in practice this is not observed in SpeakEasy. Cluster accuracy improves when labels from the last several time-steps are included in the calculation of expected and actual labels. However, initially the network has no history of labels, so we create an artificial buffer of random neighboring labels. This buffer prevents the algorithm from becoming trapped in an early equilibrium, and also provides unique initial conditions, which are useful when clustering the same dataset multiple times.

### Defining disjoint and overlapping communities

Stochastic clustering algorithms such as SpeakEasy can generate many partitions (sets of clusters) from repeated runs with different initial conditions. The ability to generate many partitions is useful because it can quantify the stability of each cluster (Fig. 5B). It is also useful in identifying multi-community nodes. We identify such nodes as those which alternate between two stable communities, when looking across many partitions (example shown in Fig. 1: node tagged with red and orange labels). However, combining multiple partitions to identify stable final clusters (consensus clustering) and to identify multi-community nodes is a challenging mathematical process, potentially even more difficult and computationally intensive than clustering individual elements^18^,^58^,^59^. While many consensus clustering techniques attempt to identify the optimal partition, and to use that as the consensus clustering result, we choose to define a final set of clusters in a way that is representative of the distribution of partitions. Specifically, the partition with the highest average adjusted Rand index (ARI) among all other partitions is selected as the representative partition. Clusters identified in this way are likely to be robust, because spurious partitions will have lower ARI scores with most other partitions. Multi-community nodes are selected as nodes which co-occur with more than one of the final clusters with greater than a user-selected frequency (see Supplementary Materials).

## Additional Information

**How to cite this article**: Gaiteri, C. *et al.* Identifying robust communities and multi-community nodes by combining top-down and bottom-up approaches to clustering. *Sci. Rep.* **5**, 16361; doi: 10.1038/srep16361 (2015).

## Supplementary Material

Supplementary Materials

## Figures and Tables

**Table 1:** Overview of datasets used in SpeakEasy community detection.

Dataset title	Network size (#nodes)	Biological scale	Data type	Cluster validation	Output	Conclusion
LFR benchmarks	1000–5000	NA	unweighted symmetric networks	known/synthetic clusters	benchmark clusters - comparable to other methods	Top recorded performance on LFR benchmarks to date
Various real networks	34–320000	NA	unweighted symmetric networks	modularity measures	cluster separation statistics - comparable to other methods	Predicted communities are well-separated
Human Brain Atlas (HBA); Cancer Cell Line Encyclopedia (CCLE)	8000–18000	gene	gene expression	Gene Ontology (GO)	co-regulated gene sets	Possible to robustly detect overlapping gene clusters
Gavin *et al.*; Collins *et al.*	700–1100	protein	AP-MS protein interactions	small-scale experiments	protein complexes and multi-community proteins	Most accurate recovery of true protein complexes to date
Immunological Genome Project (Immgen)	212	cell-type	cell type-specific gene expression	cell-surface markers	families of cell-types, at multiple resolutions	Cannonical cell type classification is mirrored in cluster results
Spike-sorting	9900	cell activity	extracellular neuron recordings	known/synthetic clusters	spikes associated with specific neurons	SpeakEasy accuratly associates spike waveforms with specific neurons
Parkinson disease rs-fMRI	264	tissue	brain resting state fMRI	permutation testing	groups of synchronized brain regions	SpeakEasy identifies disease-related changes to co-active brain regions

We test community detection across a range of biological datasets to robustly characterize the ability to define practically useful biological communities.

**Table 2:** Comparison of the abstract goodness of clustering results using modularity (Q and Q_ds_) on many types of networks between SpeakEasy and a top-performing overlapping clustering method (GANXiS).

Network	*n*	*m*	GANXiS (Q)	SpeakEasy (Q)	Percentage difference (Q)	GANXiS (Q_ds_)	SpeakEasy (Q_ds_)	Percentage difference (Q_ds_)
karate	34	78	0.3924	**0.4198**	6.75	0.2116	**0.2302**	8.42
dolphins	62	159	0.4408	**0.5017**	12.92	0.1664	**0.2378**	35.33
Les. Mis.	77	254	0.5224	**0.5480**	4.78	0.2808	**0.3438**	20.17
pol. books	105	441	0.4831	**0.4973**	2.90	0.1634	**0.2396**	37.82
football	115	613	**0.5878**	0.5811	−1.15	0.3792	**0.4856**	24.61
Santa Fe	118	200	**0.7166**	0.4792	−39.69	0.2099	**0.2963**	34.13
jazz	198	2742	0.2816	**0.4443**	44.83	0.1917	**0.2134**	10.71
railway	297	1213	**0.6989**	0.6098	−13.61	0.2632	**0.3756**	35.20
*c. elegans*	453	2525	0.1706	**0.3883**	77.90	0.05151	**0.1079**	70.75
email	1133	5254	**0.5035**	0.4916	−2.39	0.05366	**0.1025**	62.55
pol. blogs	1224	19022	**0.4177**	0.3533	−16.71	0.0230	**0.0426**	59.78
net science	1461	2742	**0.9039**	0.7657	−16.55	**0.5797**	0.3600	−46.76
PGP	10680	24316	**0.8039**	0.7315	−9.43	0.1595	**0.1906**	17.77
DBLP	260998	950059	**0.6622**	0.6066	−8.76	0.2018	**0.2628**	26.29
Amazon	319948	880215	**0.7659**	0.7094	−7.66	0.2007	**0.2556**	24.04

By testing community detection in many types of networks we can assess the quality of SpeakEasy community detection across networks with different topologies. Top modularity scores are shown in bold. “Karate” is a network of friendships between college club participants from the 1970’s. “Pol books” is a co-purchasing network of books on political topics that were published in 2004. “Netscience” is a co-citation network among network science authors. “Dolphins” is a social interaction network of a bottlenose dolphin pod from New Zealand. “Les Miserables” is a network of character interactions in the novel by Victor Hugo. “Football” is a network of American Division 1A college football teams, linked by matches. “Sante Fe” is a co-authorship network of members at the Santa Fe Institute. Links in the “Jazz” network denote musical collaborations between the years 1912 and 1940. “Pol blogs” is a network of hyperlinks among political-oriented blogs in 2005. “Email” is a network of emails linking various Enron employees. The PGP network describes Pretty Good Privacy key signing. “DBLP” is a co-authorship network in computer science, whose communities tend to be related to specific conferences or journals. “Amazon” is a network of item co-purchases.

**Table 3:** Comparison between protein complexes defined by small-scale experiments versus those inferred from high-throughput interaction datasets.

Ground truth definition source	Network dataset	SpeakEasy output type	NMI	Omega	Precision	Recall	F-score	Precision (overlapping)	Recall (overlapping)	F-score (overlapping)
CYC2008	Collins *et al.*	Disjoint	0.7237	0.6382	0.9844	0.8259	0.8982	NA	NA	NA
CYC2008	Collins *et al.*	Overlapping	0.7120	0.5961	0.9845	0.8055	0.8860	0.2151	0.1170	0.1515
CYC2008	Gavin *et al.*	Disjoint	0.4502	0.4530	0.8915	0.5395	0.6722	NA	NA	NA
CYC2008	Gavin *et al.*	Overlapping	0.4498	0.4265	0.8837	0.5292	0.6620	0.2105	0.0958	0.1317
MIPS	Collins *et al.*	Disjoint	0.6669	0.3740	0.9208	0.8701	0.8947	NA	NA	NA
MIPS	Collins *et al.*	Overlapping	0.6665	0.3880	0.9118	0.8588	0.8845	0.7821	0.1227	0.2122
MIPS	Gavin *et al.*	Disjoint	0.5155	0.2001	0.8889	0.7238	0.7979	NA	NA	NA
MIPS	Gavin *et al.*	Overlapping	0.4929	0.2259	0.9053	0.7127	0.7975	0.7143	0.2092	0.3236
SGD	Collins *et al.*	Disjoint	0.7147	0.5652	0.9597	0.7510	0.8426	NA	NA	NA
SGD	Collins *et al.*	Overlapping	0.7058	0.5106	0.9733	0.7470	0.8453	0.4766	0.2048	0.2865
SGD	Gavin *et al.*	Disjoint	0.5474	0.5215	0.9907	0.6255	0.7668	NA	NA	NA
SGD	Gavin *et al.*	Overlapping	0.5460	0.5130	0.9722	0.6175	0.7553	0.3659	0.0652	0.1107

Table values consist of normalized mutual information (NMI) between predicted and canonical protein complexes.

**Table 4:** Comparison of clusters and subclusters of gene expression vectors from sorted cell populations to canonical families of mouse immune cell-types.

	SLHC,w/predicted cluster # (2)	ALHC,w/predicted cluster # (2)	CLHC,w/predicted cluster # (2)	SLHC, w/true # tier-1 clusters (15)	ALHC, w/true # tier-1 clusters (15)	CLHC, w/true # tier-1 clusters (15)	SLHC, w/true # tier-2 clusters (23)	ALHC, w/true # tier-2 clusters (23)	CLHC, w/true # of tier-2 clusters (23)	SpeakEasy primary clusters	SpeakEasy secondary clusters	cell class (T cell, B cell etc)	tissue of origin	cell type+tissue of origin
SLHC,w/predicted cluster # (2)	1	0.0305	0.0573	0.3463	0.2075	0.0992	0.2133	0.1877	0.1764	0.264	0.0676	0.0698	0.0466	0.0829
ALHC,w/predicted cluster # (2)	0.0305	1	0.4087	0.193	0.5373	0.4803	0.5522	0.486	0.4436	0.4769	0.3075	0.4158	0.2802	0.3813
CLHC,w/predicted cluster # (2)	0.0573	0.4087	1	0.1402	0.4871	0.5189	0.4162	0.5101	0.4792	0.2844	0.2328	0.3291	0.2049	0.3153
SLHC, w/true # of tier-1 clusters (15)	0.3463	0.193	0.1402	1	0.4809	0.3305	0.6158	0.4862	0.3765	0.2494	0.3262	0.343	0.3147	0.3888
ALHC, w/true # of tier-1 clusters (15)	0.2075	0.5373	0.4871	0.4809	1	0.7836	0.7859	0.9045	0.7798	0.6381	0.5993	0.6752	0.4893	0.6733
CLHC, w/true # of tier-1 clusters (15)	0.0992	0.4803	0.5189	0.3305	0.7836	1	0.6724	0.8258	0.9236	0.5455	0.5914	0.6712	0.5094	0.678
SLHC, w/true # of tier-2 clusters (23)	0.2133	0.5522	0.4162	0.6158	0.7859	0.6724	1	0.8056	0.6808	0.5608	0.5709	0.6156	0.5554	0.6635
ALHC, w/true # of tier-2 clusters (23)	0.1877	0.486	0.5101	0.4862	0.9045	0.8258	0.8056	1	0.8316	0.5921	0.6259	0.6979	0.5575	0.7255
CLHC, w/true # of tier-2 clusters (23)	0.1764	0.4436	0.4792	0.3765	0.7798	0.9236	0.6808	0.8316	1	0.5602	0.6422	0.7312	0.5459	0.73
SpeakEasy primary clusters	0.264	0.4769	0.2844	0.2494	0.6381	0.5455	0.5608	0.5921	0.5602	1	0.4012	0.4867	0.3169	0.4885
SpeakEasy secondary clusters	0.0676	0.3075	0.2328	0.3262	0.5993	0.5914	0.5709	0.6259	0.6422	0.4012	1	0.7607	0.5485	0.7765
cell class (T cell, B cell etc)	0.0698	0.4158	0.3291	0.343	0.6752	0.6712	0.6156	0.6979	0.7312	0.4867	0.7607	1	0.5192	0.8334
tissue of origin	0.0466	0.2802	0.2049	0.3147	0.4893	0.5094	0.5554	0.5575	0.5459	0.3169	0.5485	0.5192	1	0.8099
cell type+tissue of origin	0.0829	0.3813	0.3153	0.3888	0.6733	0.678	0.6635	0.7255	0.73	0.4885	0.7765	0.8334	0.8099	1

Table values consist of normalized mutual information (NMI) between predicted and canonical protein complexes, for hierarchical clustering methods with various levels of linkage and numbers of clusters. SLHC: single-linkage hierarchical clustering; ALHC: average-linkage hierarchical clustering; CLHC: complete-linkage hierarchical clustering.
