# Supplementary material for: Identifying robust communities and multi-community nodes by combining top-down and bottom-up approaches to clustering
Source: Sci Rep. 2015 Nov 9;5:16361. doi: 10.1038/srep16361 (PMC4637843; doi:10.1038/srep16361)
Supplement: Supplementary Materials [file srep16361-s1.pdf]

**Supplemental methods for: Identifying robust communities and multi-community nodes by combining top-down and bottom-up approaches to clustering**

Chris Gaiteri[1,2*], Mingming Chen[3*], Boleslaw Szymanski[3,4], Konstantin Kuzmin[3], Jierui Xie[3*,5], Changkyu Lee[2], Timothy Blanche[2], Elias Chaibub Neto[6], Su-Chun Huang[7], Thomas Grabowski[7,8], Tara Madhyastha[8] and Vitalina Komashko[9]

1 Rush University Medical Center, Alzheimer's Disease Center, Chicago, IL
2 Allen Institute for Brain Science, Modeling, Analysis and Theory Group, Seattle, WA
3 Rennselaer Polytechnic Institute, Department of Computer Science, Troy, NY
4 Społeczna Akademia Nauk, Łódź, Poland
5 Samsung Research America, San Jose, CA
6 Sage Bionetworks, Seattle, WA
7 University of Washington, Department of Neurology, Seattle, WA
8 University of Washington, Department of Radiology, Seattle, WA
9 Trialomics, Seattle WA
*These authors contributed equally to this work

Corresponding author (Chris Gaiteri) email: gaiteri@gmail.com

## Supplemental Methods

### Data sources for synthetic and non-biological networks

*Synthetic network benchmarks*
For disjoint community detection, we used the LFR benchmarks with 1000 nodes with average degree (number of connections) of 15 and maximum degree of 50 (Figure 2A)[1]. The exponent, γ, for the degree sequence varies from 2 to 3 and the exponent, β, for the community size distribution, varies from 1 to 2. Results over four pairs of the exponents (γ, β) = (2, 1), (2, 2), (3, 1), and (3, 2) indicate community detection is robust over this parameter range (Figure 2A, Figure S1). For each of these community distributions, the fraction of between- versus within-community connections (μ) was varied from 0.05 to 0.95 (Figure 2, Figure S1). This means that each node shares a fraction (1- μ) of its edges with the nodes in its community and a fraction μ of its edges with the nodes outside its community. Thus, low μ-values indicate the test networks which are composed of relatively isolated communities, and which should be relatively easy to accurately define. To generate robust results, 10 network instances are generated for each value of μ and we report average performance across a variety of statistics commonly used to assess community recovery (Figure 2A), including NMI, ARI and F-measure[2].

When networks contain multi-community nodes, whose links are evenly divided between multiple communities, community detection is even more challenging. To

generate overlapping networks in the LFR benchmarks, we follow the standard practice of setting 10% of the nodes to have their connection evenly divided between two or more communities ($O_m$ parameter) (Figure 2C). This form of overlapping is distinct from the random between-community connections (parameterized by μ), because this 10% subset of nodes is equally well connected to multiple communities. We report average community recovery, using a variety of statistics, averaged over 10 network instances for each value of $O_m$, across a variety of community mixing levels (μ) (Figure 2D). Not all traditional community quality metrics extend to overlapping networks, so we implement an extension of the normalized mutual information (NMI) and the Omega Index (OI) to track recovery of overlapping communities[2]. The OI is equivalent to the common Adjusted Rand Index (ARI) for disjoint communities, while the overlapping NMI measure does not reduce to the standard formulation of NMI for disjoint communities.

These overlapping community detection results could primarily be driven by SpeakEasy's excellent disjoint community performance, since most nodes have a single non-overlapping true community assignment. Therefore, we specifically track recovery of multi-community nodes using a statistic we call F(multi)-score, which is computed identically to the standard F-score, but the inputs specifically track if multi-community nodes are correctly assigned to all of their true communities (Figure 2D). These results indicate that SpeakEasy fulfills the goal of detecting multi-community nodes and does not purely rely on its strong disjoint community detection abilities. Like the related label propagation algorithm, GANXiS[3], SpeakEasy shows a rare upward trend in F(multi)-score as $O_m$ increases. This result, specifically on multi-community nodes (Figure 2D), should be considered with the overall lower community recovery at higher $O_m$ values (Figure 2C). Together, these results indicate that while multi-community nodes increase the difficulty of community detection, it is still possible to cluster such networks accurately and to detect the sets of communities associated with multi-community nodes.

*Abstract clustering performance on real-world networks*

Traditionally, performance of clustering methods on networks with *unknown* correct clustering solutions is measured in terms of modularity (“Q”). Modularity measures the number of within-community connections, relative to the number expected at random[4]. This measure has a maximum value of 1, but in practice maximum possible Q-value will be less than 1.0, due to between-community links. Nevertheless, comparing Q-values between partitions generated by different clustering methods provides a relative measure of their ability to detect well-separated communities. Since the classic modularity formula does incorporate the number of between module connections but not the density of nodes within communities, anomalous situations can occur, wherein better partitions have *lower* Q values (contrary to the intended operation of Q)[5,6]. Therefore, we also track performance of SpeakEasy in terms of an updated modularity measure, $Q_{ds}$, which adds a correction term to the original modularity formula to create a more accurate metric[5].

## Algorithm details

**Algorithm 1** : SpeakEasy

**Input:** Graph $G = (V, E)$, representing the network on which a community structure is to be detected; $id_v$, an identifier of node $v$, $\forall v \in V$; $numHistoryLabels$, the number of labels in the historical label buffer of each node; $numIter$, the number of iterations to consider when determining the termination of the label propagation phase if there were no changes in label histories; $r$, the stringency threshold parameter for overlapping communities; $numRuns$, the number of runs for consensus clustering

**Output:** A set of communities $\mathcal{C} = \big\{c | c = \{v | v \in V \land v \text{ is assigned to community } c\}\big\}$; $id_c$, an identifier of community $c$, $\forall c \in \mathcal{C}$

**procedure** CONSENSUSCLUSTERING

**Initialize the co-occurrence matrix**

- $\mathcal{A} \leftarrow [\,][\,]$
- **for all** $u, v \in V$ **do**
  - $\mathcal{A}[u][v] \leftarrow 0$
- **end for**

**Run replicate community detection runs and fill in the co-occurrence matrix**

- **for** $i \leftarrow 1$ to $numRuns$ **do**
  - $\mathcal{C}_i \leftarrow$ COMMUNITYDETECTION
  - **for all** $u, v \in V$ **do**
    - $u_c \leftarrow id_c$ s.t. $c \in \mathcal{C}_i \land u \in c$
    - $v_c \leftarrow id_c$ s.t. $c \in \mathcal{C}_i \land v \in c$
    - **if** $u_c = v_c$ **then**
      - $\mathcal{A}[u][v] \leftarrow \mathcal{A}[u][v] + 1$
    - **end if**
  - **end for**
- **end for**

**Determine the final representative partition**

- **for all** $i \leftarrow 1$ to $numRuns$ **do**
  - **for all** $j \leftarrow 1$ to $numRuns$ **do**
    - $\mathcal{R}_{ij} \leftarrow \text{ARI}(\mathcal{C}_i, \mathcal{C}_j)$
  - **end for**
  - $\overline{\mathcal{R}_i} \leftarrow \frac{\sum_{j=1}^{numRuns} \mathcal{R}_{ij}}{numRuns}$
- **end for**
- $index \leftarrow \underset{i \in [1..numRuns]}{\arg\max} \ \overline{\mathcal{R}_i}$
- $\mathcal{C} \leftarrow \mathcal{C}_{index}$
- **if** $r < 1$ **then**
  - **for all** $v \in V$ **do**
    - **for all** $c \in \mathcal{C}$ s.t. $v \notin c$ **do**
      - $\overline{\mathcal{A}_{v,c}} \leftarrow \frac{\sum_{u \in c} \mathcal{A}[v][u]}{|c| \cdot numRuns}$
      - **if** $\overline{\mathcal{A}_{v,c}} > r$ **then**
        - $c \leftarrow c \cup \{v\}$
      - **end if**
    - **end for**
  - **end for**
- **end if**
- **return** $\mathcal{C}$

**end procedure**

**Algorithm 1** : SpeakEasy (continued)

```
procedure CommunityDetection
Initialize the history of labels
    N_v = {u | ∃e : u ∈ V ∧ e ∈ E ∧ e = (v, u)  (or e = {v, u}  for undirected graphs)}
    C ← ∅
    for all v ∈ V do
        L_v ← []
        LC_v ← []
        L_v[1] ← id_v
    end for
    for all v ∈ V do
        for i ← 2 to numHistoryLabels do
            if N_v ≠ ∅ then
                neighbor ←  a random node from N_v
            else
                neighbor ← v
            end if
            L_v[i] ← L_neighbor[1]
        end for
    end for
Perform label propagation
    totalNumLabels ← |V| · numHistoryLabels
    repeat
        globalFrequencies ← []
        for all v ∈ V do
            for i ← 1 to numHistoryLabels do
                l ← L_v[i]
                globalFrequencies[l] ← globalFrequencies[l] + 1/totalNumLabels
            end for
        end for
        for all v ∈ V do
            for all neighbor ∈ N_v do
                for i ← 1 to numHistoryLabels do
                    l ← L_neighbor[i]
                    if ∃LC_v[l] then
                        LC_v[l] ← LC_v[l] + 1
                    else
                        LC_v[l] ← 1
                    end if
                end for
            end for
            for all l s.t. ∃LC_v[l] do
                count_l ← LC_v[l]
                expectedCount_l ← globalFrequencies[l] · |N_v| · numHistoryLabels
            end for
            mostUnexpectedLabel ← argmax_{l s.t. ∃LC_v[l]} (count_l − expectedCount_l)
            for i ← 1 to numHistoryLabels − 1 do
                L_v[i] ← L_v[i + 1]
            end for
            L_v[numHistoryLabels] ← mostUnexpectedLabel
        end for
    until no changes in L_v, ∀v ∈ V for the last numIter iterations
```

**Algorithm 1** : SpeakEasy (continued)

**Extract communities from label histories**

**for all** $v \in V$ **do**
  $l \leftarrow \underset{l \text{ s.t. } \exists \mathcal{LC}_v[l]}{\arg\max} \ \mathcal{LC}_v[l]$
  **if** $\exists c \in \mathcal{C}$ s.t. $id_c = l$ **then**
    $c \leftarrow c \cup v$
  **else**
    $c \leftarrow \{v\}$
    $id_c \leftarrow l$
    $\mathcal{C} \leftarrow \mathcal{C} \cup c$
  **end if**
**end for**
**return** $\mathcal{C}$
**end procedure**

*Computational complexity of SpeakEasy*

The initialization takes O(|V| * *numHistoryLabels*) steps to assign an initial buffer with a certain number of history labels to each node.

The label propagation procedure requires O(|V| * *numHistoryLabels*) operations to calculate the global frequencies of all labels in the network.

Getting the actual numbers of labels that a node receives from its neighbors costs O(<k> * *numHistoryLabels*) where <k> is the average degree of the network.

The procedure to determine the most unexpected label for a node has a complexity at most O(<k> * *numHistoryLabels*) because there are at most *numHistoryLabels* distinct labels in the buffer of each of its neighbors.

Thus, it totally requires O(|V| * <k> * *numHistoryLabels*) to update each node's historical label buffer with the most unexpected popular label received from its neighbors. In total, the label propagation procedure has a complexity of O(|V| * <k> * *numHistoryLabels*) which can be expressed as O(|E| * *numHistoryLabels*). Moreover, the label propagation takes a certain number of iteration, denoted as *T*, which is usually a small constant.

Thus, the complexity for the label propagation of SpeakEasy is O(*T* * |E| * *numHistoryLabels*). Since *T* and *numHistoryLabels* are small constants (*T* has a default value of 50 and *numHistoryLables* defaults to 5), the overall complexity can be reduced to O(|E|) which is linear in terms of the network size for sparse matrices.

*Computational complexity of SpeakEasy in practice*

SpeakEasy scales linearly with the number of edges, and can cluster typical biological networks quickly. For instance SpeakEasy clusters 10,000 nodes with random 2% connectivity density (2 million links) in ~10 seconds with an i7 2620M processor, using ~1GB of RAM. Clustering the amazon co-purchase network with over 300,000 nodes (Table 2) required 45 seconds on the same processor and 0.5GB of RAM. The typically sparse connectivity of biological networks[7] in combination with the linear complexity of SpeakEasy make it feasible to derive consensus clustering estimates, by clustering networks many times using differential initial conditions, to identify stable communities

and multi-community nodes. Even on large full matrices, such as coexpression networks, the efficiency of SpeakEasy enables stable community estimates using typical hardware.

*Selecting final partitions and defining multi-community nodes:*
Stochastic clustering techniques such as SpeakEasy, have the potential to generate more accurate and robust results than typical methods that output a single set of communities[8,9]. Because SpeakEasy generates many "partitions" (sets of communities) during replicate runs, we are faced with the task of combining these outputs into a final partition. This process is known as consensus clustering and in many cases solutions to this problem are more statistically challenging than basic clustering, essentially because it involves relationships of semi-overlapping sets. The manner in which the "best" final partition is chosen among many stochastically generated partitions is independent of the method used to generate the partitions. Therefore, improving consensus clustering could lead to better SpeakEasy results.

Overlapping communities and multi-community nodes are defined through a combination of the final representative partition, and the co-occurrence matrix, which is generated by all partitions. The final representative partition is chosen to be the one with the highest average ARI with all other partitions. Individual nodes with significant co-occurrence weight in more than one community can be classified as multi-community nodes. Each entry A(i,j) of the co-occurrence matrix denotes how many times nodes *i* and *j* cluster together, under replicate SpeakEasy partitions. We set the threshold for community membership as a function of the maximum number of community memberships considered realistic in a particular biological setting, specifically 1/max-number-of-communities. This adaptive threshold is intuitive, because as the number of communities associated with a given node increases, the frequency in which a node is found in any one of the communities decreases. This threshold is used in conjunction with the co-occurrence matrix: nodes that co-occur in multiple communities with an average weight (across all members of that community) greater than the threshold are identified as multi-community nodes. More or less stringency in this threshold provides control over the number of multi-community nodes. This flexibility in defining multi-community nodes is useful, because in some experimental settings it might be desirable to obtain more or less conservative definitions of multi-community nodes.

## Application-specific methods and data acquisition

*Protein-protein interaction network processing*
Protein complexes were downloaded from various databases such as MIPS and these gold-standard complexes were compared to communities derived from binary interactions identified in the Gavin et al.[10] and Collins et al.[11]. Because we validate the clustering output by comparing to ground truth complexes, we operate on the (large) subsets of Gavin and Collins networks wherein the nodes on both sides of a given link

are present in the ground truth.  If we did not do this, there would be nodes in the network for which we do not have ground truth community information.

*Application to sorted cell type populations*

We compare SpeakEasy results to several hierarchical clustering techniques that are often “first pass” clustering methods applied to cell type datasets such as Immgen. While hierarchical techniques generate multiple levels of communities, it is unclear where to “cut” the hierarchical “tree” of communities in order to extract optimum communities and/or subcommunities.  Because the optimal number of communities in a dataset is generally unknown, we estimate the number of cell types in Immgen, using ten different methods[12].  When applied to Immgen, most of these methods proposed a single community containing all cells, or placed every cell in its own community. Therefore, we used the mode of estimates that do not fall at these extremes (recommendation: two communities).  When using the recommended number of communities, all forms of hierarchical clustering showed relatively low correspondence with known cell types.  We also test results when hierarchical methods are supplied with the true number of communities (information that is rarely available).  Also, we compare results from single, average and complete hierarchical linkage methods.  When compared to known cell classes (B cell, natural killer cells etc) SpeakEasy communities show higher NMI with the standard Immgen definitions than any hierarchical method, even when those methods are supplied with the true number of communities.  The second iterative application of SpeakEasy also shows higher NMI with the nested classification of cell class with tissue of origin.  In all cases it is much more accurate than when using the predicted number of true communities, which is a more realistic scenario.  Moreover, results from single versus average or complete linkage vary substantially in their ability to recover true communities, but for a typical dataset where the true communities are unknown, the best linkage method is rarely known in advance.

*Gene coexpression network generation and assessment*

Because communities containing thousands of genes are difficult to test experimentally, we apply SpeakEasy iteratively three times to these datasets to reduce the typical community size to a few hundred genes, which is experimentally tractable.  This might seem at odds with SpeakEasy’s ability to automatically select community number. However, unlike hierarchical clustering, SpeakEasy cannot be forced to output subcommunities, because it is always admissible for the algorithm to place all nodes into a single community.  To calculate median Bonferroni-corrected p-values for functional enrichment, we consider the top-scoring category for each module in a gene ontology biological process, among all modules with at least 30 genes (which is a practical threshold for GO enrichment) with at least one category scoring an un-corrected enrichment score of $p<.01$.

The weighted gene networks coexpression network analysis (WGNCA) tool is frequently used to identify coexpressed gene sets[13,14].  There are a significant number of practical and performance measures that distinguish SpeakEasy from this older method.  For instance, generating communities with WGCNA entails (i) fitting a scale-free distribution, which may not be appropriate for all datasets, (ii) filtering link weights, and then (iii)

performing a hierarchical treecut to select communities, a process which is notoriously sensitive to noise[15]. Indeed a supplementary routine for WGCNA with 19 parameters is offered to manually "tune" the hierarchical treecut for good results[16]. On the other hand, for this application to coexpression networks, SpeakEasy does not require any link filtering or distribution fitting and uses just a single parameter (number of times to sub-cluster). Furthermore, SpeakEasy gene sets are backed by the best recorded performance on the LFR benchmarks. In comparison, WGNCA cannot even be tested on the standard LFR benchmarks because it has so many manual tuning requirements. Functional enrichment scores for the top ten most enriched gene sets are either identical or highly similar for both methods, in both HBA and CCLE datasets, with larger differences in functional enrichment for smaller modules, with overall lower enrichment scores. The median Bonferroni-corrected p-values for functional enrichment across all modules are of equal magnitude on the HBA dataset, while they are 3-9 orders of magnitude more enriched for SpeakEasy communities derived from CCLE.

*Electrophysiology methods*

To simulate multichannel neural recordings from which to extract action-potentials (spikes) to test spike sorting, previously sorted spike waveforms[17] were added to brain noise (300-7kHz bandpass, 6.92uV RMS) to create a simulated 54 channel time series from a depth electrode array (spanning ~1mm with sites spaced every ~50um). 43 unique spike samples were added to the noise file 225 times (i.e. generating 43 communities of 225 elements, as well as one pseudo-community of 225 elements composed purely of noise). The waveform around each spike consists of a 1ms window (25 sampled time points) centered around the peak amplitude. A null class was included to simulate the presence of spurious spike detections that may occur when extracting spike waveform samples. We find that while the primary clusters detected by SpeakEasy provide good results, they can be improved further by applying it to each cluster, to detect additional subclusters (Table S1). Additional iterative subclustering does not improve results, indicating that only one round of subclustering is necessary for maximum accuracy.

*fMRI processing*

This analysis includes 27 subjects with PD ($M_{age}$= 66.52) and 21 controls ($M_{age}$ = 62), selected from a larger study[18]. Potential participants were excluded if they had a history of any primary neurodegenerative disease other than idiopathic Parkinson Disease, a history of brain surgery for PD, moderate to severe dyskinesia, significant head trauma, stroke history, severe or unstable cardiovascular disease, contraindications to MRI, or a Montreal Cognitive Assessment score (MoCA)[19] lower than 23. This study was approved by the University of Washington Institutional Review Board. All participants provided written informed consent.

See Table S2 for sample characteristics. Participants were predominantly right-handed. PD patients did not differ significantly from controls on age, education, or scores on the MoCA. The PD subjects have declined from their premorbid cognitive abilities, but they are well within the range of normative values for normal controls[19]. PD subjects had significantly higher scores on the UPDRS motor subscale[20].

Males were over-represented in the PD group, consistent with higher incidence rates of Parkinson disease in men[21]. PD patients ranged from Hoehn and Yahr stage 1 to 2.5 with most at stage 2 (N=17). At the time of the scan and corresponding neuropsychological evaluations, most PD patients were taking dopaminergic medications (24% were taking both levodopa and a dopamine agonist, 38% were taking only levodopa, 19% were taking only a dopamine agonist, and 19% were taking no dopaminergic medications).

*MRI acquisition*

Scans were performed after morning doses of dopaminergic medication (if applicable). Data were acquired using a Philips 3T Achieva MR System (Philips Medical Systems, Best, Netherlands, software version R2.6.3) with a 32-channel SENSE head coil. During each session, whole-brain axial echo-planar images (43 sequential ascending slices, 3 millimeter isotropic voxels, field of view = 240x240x129 , repetition time = 2400 ms, echo time = 25 ms, flip angle = 79°, SENSE acceleration factor = 2) were collected parallel to the AC-PC line for a single resting state run and six task runs. Run duration was 300 volumes (12 minutes) for the resting state run which we split into two volumes of 6 minutes from each patient. The rationale for this was to enhance our ability to avoid temporally smearing among evolving brain region communities while remaining within the resting-state paradigm. A sagittal T1-weighted 3D MPRAGE (176 slices, matrix size = 256 x 256, inversion time = 1100 ms, turbo-field echo factor = 225, repetition time = 7.46 ms, echo time = 3.49 ms, flip angle =7°, shot interval = 2530 ms) with 1 mm isotropic voxels was also acquired for registration.

*MRI processing*

Functional images from rest or task were processed identically using a pipeline developed using software from FSL[22], FreeSurfer[23], and AFNI[24]. Data were corrected for motion using FSL MCFLIRT (M. Jenkinson, Bannister, Brady, & Smith, 2002). The pipeline removed spikes using AFNI, performed slice timing correction using FSL, and regressed out time series motion parameters and the mean signal for eroded (1mm in 3D) masks of the lateral ventricles and white matter (derived from running FreeSurfer on the T1-weighted image). We did not regress out the global signal. We did not perform bandpass filtering to avoid artificially inflating correlations or inducing structure that was not actually present in the data, and because resting state networks exhibit different levels of phase synchrony at different frequencies (Handwerker, Roopchansingh, Gonzalez-Castillo, & Bandettini, 2012; Niazy, Xie, Miller, Beckmann, & Smith, 2011). Three dimensional spatial smoothing was performed using a Gaussian kernel with a FWHM of sigma=3mm. Co-registration to the T1 image was performed using boundary based registration based on a white matter segmentation of the T1 image (epi_reg in FSL).

We selected 264 MNI coordinates from a previous partitioning of fMRI data into functional nodes by Power et al.[25]. For each coordinate, we created a 10mm diameter mask in standard space and transformed that to subjects' native space to calculate mean subject-specific timecourses for each ROI. We calculated the Pearson correlation between each pair of nodes to obtain an edge weight, representing the strength of connectivity.

*Significance of connectivity changes*

Significance of changes in community membership between resting state networks from the control cohort compared to the Parkinson's cohort were estimated through permutation tests. We (repeatedly) randomly mix control and Parkinson's samples to generate two average connectivity matrices as pseudo-disease and pseudo-control groups. There should be no disease-related differences between these matrices generated from randomized disease and control data. We generate such mixed disease+control connectivity matrices 1000 times and detect communities in each case, generating 2000 paired (pseudo-disease/pseudo-control) co-occurrence matrices. The distribution of differences between these co-occurrence matrices are used to generate a null distribution used to estimate the significance of changes within and between communities between the real control and PD co-occurrence matrices.

We compare these clustering results to those generated by Infomap[26], a clustering algorithm based on data compression that has previously been used in fMRI analysis [25]. Infomap cannot take advantage of negative links found in correlations matrices, so we follow the standard practice of converting all links to positive values. We find that Infomap places all brain regions into a single community, unless links are extensively filtered. After correlations with R-value < 0.75 are removed, communities begin to emerge. However, these communities are not robust; for instance, the difference between various Infomap-based partitions (at different correlation thresholds) is as large as the difference between the control and PD partitions, using SpeakEasy (Table S3). Furthermore, Infomap communities are a point estimate, and there is no measure of the robustness of communities at a particular threshold.

**Figure S1 Robust clustering performance with various community size distributions and intra-community degree distributions.** (A) Various disjoint community recovery metrics for networks from LFR benchmarks with n=1000, $\gamma$ (community size distribution) =3, $\beta$ (within-community degree distribution) =2. (B) Disjoint community recovery metrics for networks from LFR benchmarks with n=1000, $\gamma$=3, $\beta$=1 (C) Disjoint community recovery metrics for networks from LFR benchmarks with n=1000, $\gamma$=2, $\beta$=2.

**Figure S2 Brain region communities detected from control subject resting-state fMRI.** The order of communities 1-6 corresponds to the order of communities shown in Figure 5. Location of brain regions in each cluster was/were visualized with the BrainNet Viewer[27].

**Table S1. Comparison of communities of similar neuronal spikes vs known spike communities.**

**Table S2. Summary of demographics of control and PD cohorts in resting-state fMRI study.**

**Table S3. Comparison of brain region communities detected in control or PD cohorts using SpeakEasy or Infomap (the later using various thresholds for link significance).**

Figure S1

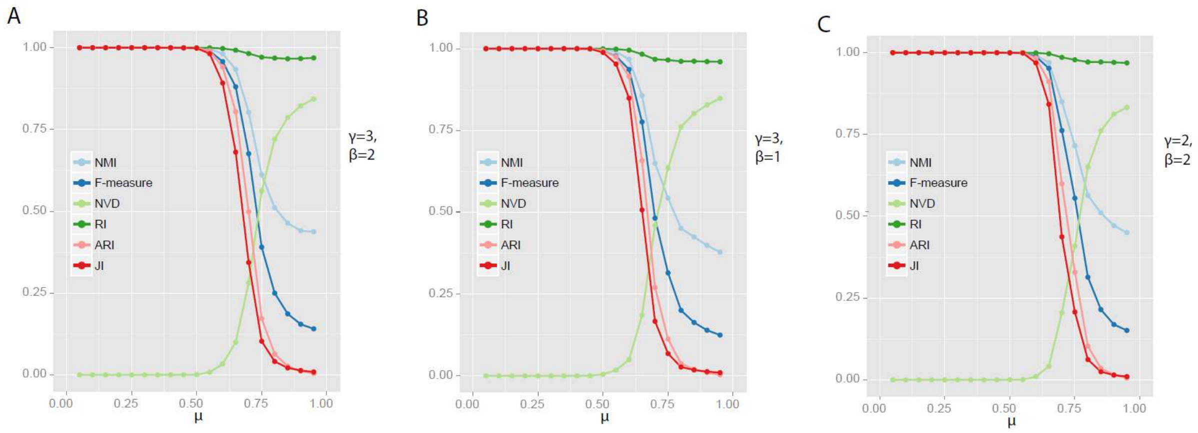

Figure S2

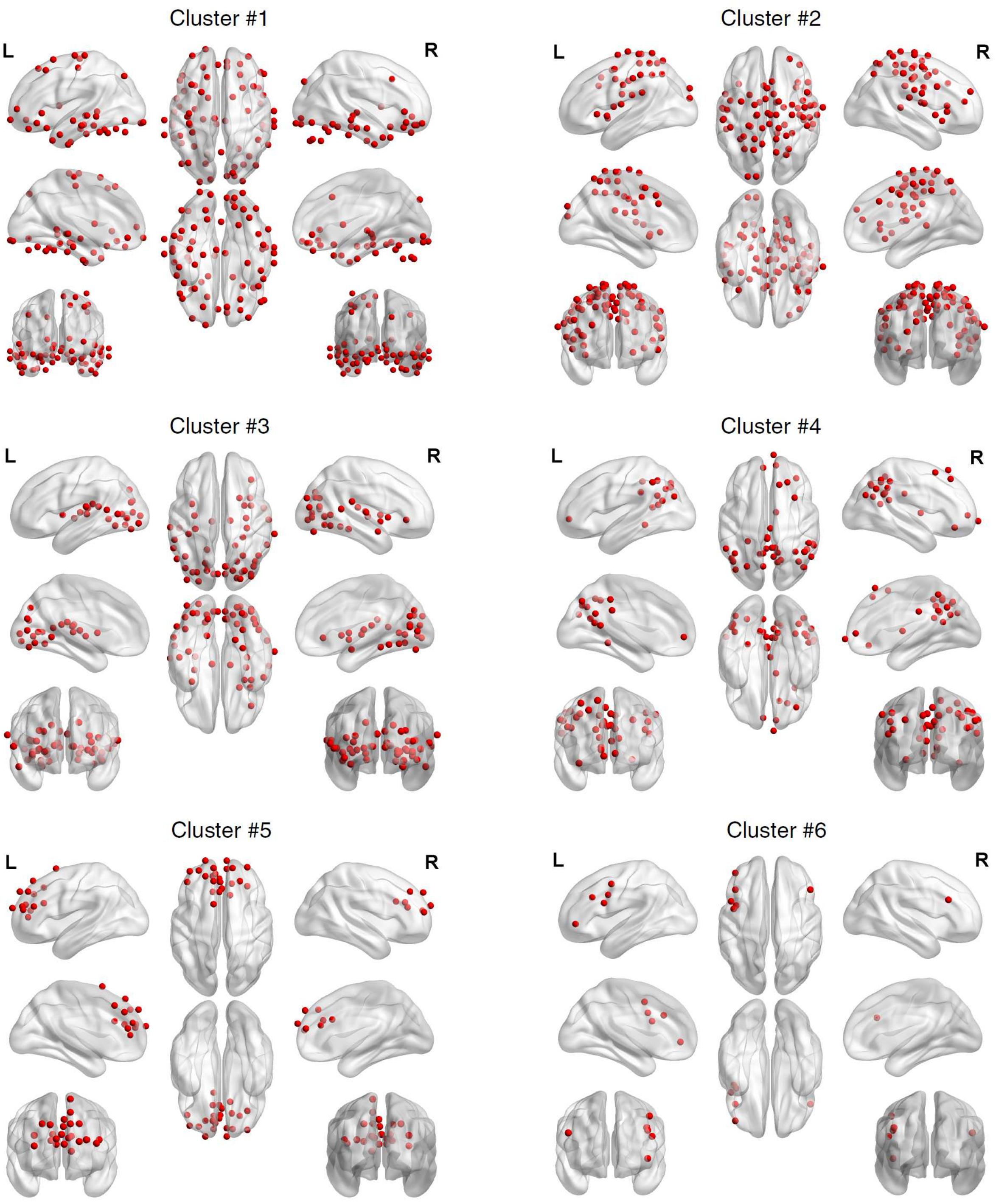
Cluster #1
L
R
Cluster #2
L
R
Cluster #3
L
R
Cluster #4
L
R
Cluster #5
L
R
Cluster #6
L
R

**Table S1**

| NMI | SpeakEasy primary communities | SpeakEasy secondary communities | SpeakEasy tertiary communities |
|---|---|---|---|
| spike waveform dataset 1 | 0.6381 | 0.8328 | 0.8085 |
| spike waveform dataset 2 | 0.6250 | 0.8202 | 0.7793 |
| spike waveform dataset 3 | 0.6127 | 0.8178 | 0.8052 |
| mean | 0.6253 | 0.8236 | 0.7977 |

| adjusted Rand index | SpeakEasy primary communities | SpeakEasy secondary communities | SpeakEasy tertiary communities |
|---|---|---|---|
| spike waveform dataset 1 | 0.6455 | 0.8274 | 0.8103 |
| spike waveform dataset 2 | 0.5981 | 0.7922 | 0.7772 |
| spike waveform dataset 3 | 0.6487 | 0.8251 | 0.8066 |
| mean | 0.6308 | 0.8149 | 0.7980 |

**Table S2**

| | **PD** | **Control** | **Total** |
|---|---|---|---|
| N | 27 | 21 | 48 |
| Age at Scan | 66.52( 9.86) | 61.90(10.00) | 64.50(10.08) |
| Sex(number males) | 20(74%) | 9 (43%) | 29 (60%) |
| Education (years) | 16.35(2.10) | 15.90(2.39) | 16.15(2.22) |
| Hoen & Yahr | 2.03 (1–2.5) | | |
| Handedness (Right) | 23 | 19 | 42 |
| Dominant side of motor symptoms | 7 Left/ 18 Right/ 2 Symmetric | | |
| UPDRS Part I | 10.00(5.51) | | 10.00(5.51) |
| UPDRS Part II | 8.81(5.35) | | 8.81(5.35) |
| UPDRS Part III | 23.30( 8.49) | 0.81( 1.40) | 13.46(12.95) |
| UPDRS Part IV | 1.85(3.59) | | 1.85(3.59) |
| Levodopa (current) | 18 | 0 | 18 |
| Dopamine agonist (current) | 11 | 0 | 11 |
| Years since symptom onset | 8.71(5.01) | | |
| MOCA | 26.44(2.06) | 27.29(1.95) | 26.81(2.04) |
| Hopkins Verbal Learning Test | 24.48(5.69) | | |
| Golden Stroop (total correct) | 189.26(24.99) | | |
| Trails B (seconds) | 74.42(31.53) | | |

## Table S3

| NMI between partitions | Control SpeakEasy | PD SpeakEasy | Control Infomap >0.75 | Control Infomap >0.80 | Control Infomap >0.85 | Control Infomap >0.90 | Control Infomap >0.95 | PD Infomap >0.75 | PD Infomap >0.80 | PD Infomap >0.85 | PD Infomap >0.90 | PD Infomap >0.95 |
|---|---|---|---|---|---|---|---|---|---|---|---|---|
| Control SpeakEasy | 1 | 0.5141 | 0.1218 | 0.3285 | 0.5376 | 0.5952 | 0.604 | 0.0866 | 0.2319 | 0.4426 | 0.4863 | 0.5057 |
| PD SpeakEasy | 0.5141 | 1 | 0.0701 | 0.3552 | 0.4569 | 0.5897 | 0.6491 | 0.0609 | 0.3165 | 0.6957 | 0.6919 | 0.633 |
| Control Infomap >0.75 | 0.1218 | 0.0701 | 1 | 0.2122 | 0.144 | 0.1133 | 0.0989 | 0.4727 | 0.1506 | 0.094 | 0.0912 | 0.0974 |
| Control Infomap >0.80 | 0.3285 | 0.3552 | 0.2122 | 1 | 0.6043 | 0.3834 | 0.3542 | 0.108 | 0.5029 | 0.3618 | 0.3586 | 0.3266 |
| Control Infomap >0.85 | 0.5376 | 0.4569 | 0.144 | 0.6043 | 1 | 0.6502 | 0.5291 | 0.0829 | 0.3364 | 0.5114 | 0.4686 | 0.473 |
| Control Infomap >0.90 | 0.5952 | 0.5897 | 0.1133 | 0.3834 | 0.6502 | 1 | 0.702 | 0.1368 | 0.2527 | 0.6387 | 0.6605 | 0.6829 |
| Control Infomap >0.95 | 0.604 | 0.6491 | 0.0989 | 0.3542 | 0.5291 | 0.702 | 1 | 0.1225 | 0.3306 | 0.6676 | 0.7141 | 0.808 |
| PD Infomap >0.75 | 0.0866 | 0.0609 | 0.4727 | 0.108 | 0.0829 | 0.1368 | 0.1225 | 1 | 0.3245 | 0.1988 | 0.1929 | 0.1626 |
| PD Infomap >0.80 | 0.2319 | 0.3165 | 0.1506 | 0.5029 | 0.3364 | 0.2527 | 0.3306 | 0.3245 | 1 | 0.4899 | 0.4753 | 0.371 |
| PD Infomap >0.85 | 0.4426 | 0.6957 | 0.094 | 0.3618 | 0.5114 | 0.6387 | 0.6676 | 0.1988 | 0.4899 | 1 | 0.8803 | 0.7164 |
| PD Infomap >0.90 | 0.4863 | 0.6919 | 0.0912 | 0.3586 | 0.4686 | 0.6605 | 0.7141 | 0.1929 | 0.4753 | 0.8803 | 1 | 0.7559 |
| PD Infomap >0.95 | 0.5057 | 0.633 | 0.0974 | 0.3266 | 0.473 | 0.6829 | 0.808 | 0.1626 | 0.371 | 0.7164 | 0.7559 | 1 |

## References


1 Bulut, E. & Szymanski, B. K. Constructing Limited Scale-Free Topologiesover Peer-to-Peer Networks. *Parallel and Distributed Systems, IEEE Transactions on* **25**, 919-928 (2014).

2 Chen, M., Kuzmin, K. & Szymanski, B. K. Community Detection via Maximization of Modularity and Its Variants. *IEEE Transactions on Computational Social Systems* (2014).

3 Xie, J., Szymanski, B. K. & Liu, X. in *Data Mining Workshops (ICDMW), 2011 IEEE 11th International Conference on.* 344-349 (IEEE).

4 Newman, M. E. Modularity and community structure in networks. *Proceedings of the National Academy of Sciences* **103**, 8577-8582 (2006).

5 Chen, M., Nguyen, T. & Szymanski, B. K. A new metric for quality of network community structure. *HUMAN* **2**, pp. 226-240 (2013).

6 Fortunato, S. & Barthelemy, M. Resolution limit in community detection. *Proceedings of the National Academy of Sciences* **104**, 36-41 (2007).

7 Leclerc, R. D. Survival of the sparsest: robust gene networks are parsimonious. *Molecular systems biology* **4** (2008).

8 Vega-Pons, S. & Ruiz-Shulcloper, J. A survey of clustering ensemble algorithms. *International Journal of Pattern Recognition and Artificial Intelligence* **25**, 337-372 (2011).

9 Ghosh, J. & Acharya, A. Cluster ensembles. *Wiley Interdisciplinary Reviews: Data Mining and Knowledge Discovery* **1**, 305-315 (2011).

10 Gavin, A.-C. *et al.* Proteome survey reveals modularity of the yeast cell machinery. *Nature* **440**, 631-636 (2006).

11 Collins, S. R. *et al.* Toward a comprehensive atlas of the physical interactome of Saccharomyces cerevisiae. *Molecular & Cellular Proteomics* **6**, 439-450 (2007).

12 Charrad, M., Ghazzali, N., Boiteau, V. & Niknafs, A. NbClust: An R Package for Determining the Relevant Number of Clusters in a Data Set. *Journal of Statistical Software* **61**, 1-36 (2014).

13 Langfelder, P. & Horvath, S. WGCNA: an R package for weighted correlation network analysis. *BMC Bioinformatics* **9**, 559, doi:10.1186/1471-2105-9-559 (2008).

14 Zhang, B. *et al.* Integrated Systems Approach Identifies Genetic Nodes and Networks in Late-Onset Alzheimer's Disease. *Cell* **153**, 707-720, doi:10.1016/j.cell.2013.03.030 (2013).

15 Xu, R. & Wunsch, D. Survey of clustering algorithms. *Neural Networks, IEEE Transactions on* **16**, 645-678 (2005).

16 Langfelder, P., Zhang, B. & Horvath, S. Defining clusters from a hierarchical cluster tree: the Dynamic Tree Cut package for R. *Bioinformatics* **24**, 719-720 (2008).
17 Blanche, T. J., Spacek, M. A., Hetke, J. F. & Swindale, N. V. Polytrodes: high-density silicon electrode arrays for large-scale multiunit recording. *Journal of Neurophysiology* **93**, 2987-3000 (2005).
18 Madhyastha, T. M., Askren, M. K., Boord, P. & Grabowski, T. J. Dynamic Connectivity at Rest Predicts Attention Task Performance. *Brain Connect*, doi:10.1089/brain.2014.0248 (2014).
19 Nasreddine, Z. S. *et al.* The Montreal Cognitive Assessment, MoCA: a brief screening tool for mild cognitive impairment. *Journal of the American Geriatrics Society* **53**, 695-699 (2005).
20 Goetz, C. G. *et al.* Movement Disorder Society-sponsored revision of the Unified Parkinson's Disease Rating Scale (MDS-UPDRS): Scale presentation and clinimetric testing results. *Movement disorders* **23**, 2129-2170 (2008).
21 Wooten, G., Currie, L., Bovbjerg, V., Lee, J. & Patrie, J. Are men at greater risk for Parkinson's disease than women? *Journal of Neurology, Neurosurgery & Psychiatry* **75**, 637-639 (2004).
22 Jenkinson, M., Beckmann, C. F., Behrens, T. E., Woolrich, M. W. & Smith, S. M. Fsl. *Neuroimage* **62**, 782-790 (2012).
23 Fischl, B. & Dale, A. M. Measuring the thickness of the human cerebral cortex from magnetic resonance images. *Proceedings of the National Academy of Sciences* **97**, 11050-11055 (2000).
24 Cox, R. W. AFNI: software for analysis and visualization of functional magnetic resonance neuroimages. *Computers and Biomedical research* **29**, 162-173 (1996).
25 Power, J. D. *et al.* Functional network organization of the human brain. *Neuron* **72**, 665-678 (2011).
26 Rosvall, M. & Bergstrom, C. T. Maps of random walks on complex networks reveal community structure. *Proc Natl Acad Sci U S A* **105**, 1118-1123, doi:10.1073/pnas.0706851105 (2008).
27 Xia, M., Wang, J. & He, Y. BrainNet Viewer: a network visualization tool for human brain connectomics. *PLoS ONE* **8**, e68910 (2013).
